# Neuron soma size and density measurements in rat striatal regions disaggregated by sex and estrous cycle phase

**DOI:** 10.1007/s00429-025-02995-5

**Published:** 2025-08-06

**Authors:** Nathan J. Dale, Jinyan Cao, David M. Dorris, Ashtin B. Crawford, John Meitzen

**Affiliations:** 1 Department of Biological Sciences, North Carolina State University, Campus Box 7617, Raleigh, NC 27695-7617 USA; 2 Comparative Medicine Institute, North Carolina State University, Raleigh, NC USA; 3 Center for Human Health and the Environment, North Carolina State University, Raleigh, NC USA

**Keywords:** Sex, Neuron, Striatum, Sexual dimorphism, Morphometry, Estrous cycle

## Abstract

In the adult mammalian nervous system, sex differences can be manifested independently or in concert with sex-specific hormone cycles, such as the rat estrous cycle. Biological sex and related cycles influence neuronal properties in many brain regions, including the striatum, encompassing the nucleus accumbens (NAc) core, NAc shell, and caudate-putamen (CPu). While neuron soma size and density are commonly assessed in the context of biological sex, these attributes have never been investigated in the striatal regions of adult gonad-intact rodents disaggregated by sex and estrous cycle phase. Thus, we tested the hypothesis that neuron soma size and density would vary by striatal region, sex, and estrous cycle phase. Neuron soma size and density were measured in NAc core, NAc shell, and CPu from adult male rats and female rats in diestrus, proestrus, and estrus phases. Overall, neuron soma size was larger in the CPu than the NAc core and shell. Neuron density was greatest in the NAc shell, followed by the NAc core and CPu. Regarding sex, soma size was larger in male than female NAc shell and did not differ in other regions. Soma density did not sexually differ. Neither soma size nor density differed across estrous cycle phases. These results provide, for the first time, striatal neuron size and density measurements disaggregated by sex and estrous cycle phase and an indication of a sex difference in NAc shell soma size. In contrast, the estrous cycle appears to influence striatal function via other mechanisms than neuronal soma attributes.

## Introduction

Neuroscience research historically exhibits an overreliance on male animals, especially in rodent preclinical studies (Lee et al. 2025; Shansky et al. 2024; Mamlouk et al. 2020; Will et al. 2017; Beery and Zucker 2011). As the influence of biological sex and related attributes upon the nervous system has slowly become better studied with more sophisticated tools, it has become apparent that sex differences in neuronal properties can be manifested either independently or in concert with sex-specific hormone cycles. Examples of sex-specific hormone cycles include the adult female human menstrual cycle and the homologous rat estrous cycle, which prepare the brain and body for reproductive-related activities (Itriyeva 2022; Schwartz 2000). The estrous cycle heavily influences select neural substrates, including both physiological and anatomical aspects (Blume et al. 2017; Clemens et al. 2019; Proaño et al. 2024; Rocks et al. 2022; Woolley et al. 1990). In the striatal brain regions focused upon in this study, the nucleus accumbens (NAc) shell, NAc core, and caudate-putamen (CPu), sex and the estrous cycle have been shown to influence both neuron physiology as well as dendritic spine attributes, as well as many other facets (Beeson and Meitzen 2023; Catalfio et al. 2023; DePoy et al. 2024; Kniffin and Briand 2024; Lewitus et al. 2024; Proaño et al. 2018, 2020; Willett et al. 2020). Importantly, sex differences in neuron physiology and anatomy can manifest independently of the estrous cycle: whether induced via the organizational actions of hormones during a critical period, interactions with environmental, neuroimmune and/or other factors, and/or mediated directly from sex chromosomes (Arnold 2017; Mauvais-Jarvis et al. 2020; McCarthy 2020; McCarthy et al. 2015; Wade and Arnold 2004), including in the striatal regions (Cao et al. 2016; Chen et al. 2009; Dorris et al. 2015; Forlano and Woolley 2010; Willett et al. 2020; Wissman et al. 2012).

Regarding the striatal regions, soma size and density have yet to be quantified in gonad-intact adult rats disaggregated by sex and estrous cycle phase. Neuron soma size and density are important attributes to assess because they directly influence neuron and brain region function. For example, changes in neuron soma size and overall morphology can alter important functional parameters, including capacitance and excitability (Zhu et al. 2016). It has long been documented that soma size and density are cellular attributes sensitive to sex and related hormone impacts. Examples brain regions showing sex differences in neuron density include the spinal nucleus of the bulbocavernosus (SNB) (Marc Breedlove and Arnold 1981), the sexually dimorphic nucleus of the preoptic area (SDN-POA) (Gorski et al. 1980), and telencephalic song control nuclei HVC and RA in sexually dimorphic songbirds (Nottebohm and Arnold 1976; Wade and Arnold 2004). Regarding the striatal regions, it is unknown if striatal neuron density differs by sex or estrous cycle phase, and to our knowledge no brain region has been assessed for differences in neuron soma density across the estrous cycle phase. Changes in neuron density in the striatum are possible due to reports of neurogenesis in this region (Inta et al. 2015). One previous study of the striatal regions assessing soma size and density only employed gonadectomized rodents (Meitzen et al. 2011). Other studies in gonad-intact rodents assessed dendritic spines, overall region volume, physiological attributes, or did not address sex as an experimental variable (Beeson and Meitzen 2023; Campi et al. 2013; Keller et al. 2018; Wong et al. 2016; Meitzen et al. 2018).

Our research directly addresses this gap in knowledge by measuring striatal soma size and neuron density for the first time in gonad-intact animals identified by sex and estrous cycle phase. Specifically, we tested the hypothesis that neuron soma size and density would vary by striatal region, sex, and estrous cycle phase. To test our hypothesis, we first assessed soma size and density for each striatal region in male and female adult rats. These measurements allowed global comparisons between each region irrespective of sex and estrous cycle phase to validate and extend previous studies (Meitzen et al. 2011; Meredith et al. 1992). We next tested for general sex differences in each striatal region. Additionally, we also tested differences between males and females disaggregated by estrous cycle phase. Overall, our research aims to address whether or not rodent NAc shell, NAc core, and CPu exhibit sex and/or estrous cycle differences as well as providing the first quantification of rat striatal neuron soma attributes across region, sex, and estrous cycle phase.

## Methods

### Animals

All animal protocols were approved by the Institutional Animal Care and Use Committees at North Carolina State University. All animals were housed in a temperature and light-controlled room (23 °C, 40% humidity, 12:12-h light-dark cycle with lights turned on and off at 7 AM and 7 PM, respectively) at the Biological Resource Facility of North Carolina State University. All cages were polysulfone Bisphenol A (BPA) free and were filled with bedding manufactured from virgin hardwood chips (Beta Chip; NEPCO, Warrensburg, NY) to avoid endocrine disruptors present in corncob bedding (Landeros et al. 2011; Mani et al. 2005; Markaverich et al. 2002). Soy protein-free rodent chow (2020X; Teklad, Madison, WI) and glass bottle-provided water were available *ad libitum*. Female and male Wistar rats were born from timed-pregnant females purchased from Charles River Laboratories. Rats were housed with their littermates and dam until weaning. After weaning at postnatal day (PND) 21, males (*n* = 8) and females (*n* = 24) were group housed (3–4) by sex until experimental use in adulthood (PND 161.1 ± 19.11). Age of animal did not differ by experimental group (F_(3,28)_ = 2.182, *P* > 0.05). Estrous cycle was assessed in females and experimental sample size (N) was balanced across groups (diestrus phase *n* = 8, proestrus phase *n* = 8, estrus phase *n* = 8, males *n* = 8). Briefly, females were vaginally swabbed with potassium phosphate buffer solution daily in the afternoon (~ 3:00–5:00 PM). The proestrus phase assessed here corresponded with the “proestrus PM” or “late proestrus” phase as previously investigated (Beeson and Meitzen 2023). Samples were dabbed onto slides to create a wet mount preparation and visualized under a Leica DM750 light microscope with a 10x objective to determine estrous cycle phase according to cell morphology following established protocols (Proaño et al. 2020; Westwood 2008; Marcondes et al. 2002). Briefly, cellular morphology associated with the proestrus phase demonstrated nucleated epithelial cells, while cellular morphology associated with the estrous phase exhibited un-nucleated cornified cells. For the diestrus phase, leukocytes dominated the cellular morphology. Sacrifice occurred within one hour of vaginal swab in the late afternoon. This schedule was adopted to standardize handling across all animal groups to that experienced by animals in proestrus PM phase to minimize circadian rhythm influences (Butler-Struben et al. 2022) and maximize hormonal impacts; this method was an adaptation of methods from the previous study (Beeson and Meitzen 2023).

### Brain histology

**Fig. 1:**
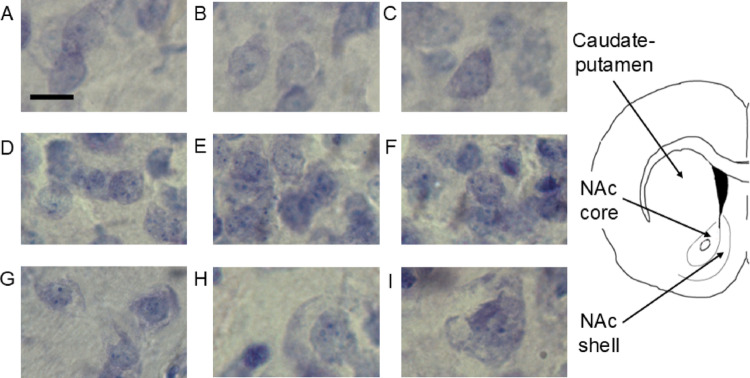
Representative images of Nissl stained striatal neurons from the NAc core, NAc shell, and CPu, illustrating differences detected between each region irrespective of sex and estrous cycle phase. Left: **A–C** NAc core neurons exhibit intermediate soma size and density compared to other regions. **D–F** NAc shell neurons show smallest soma size and highest density compared to other regions. **G–I** CPu neurons demonstrate largest soma size and lowest density compared to other regions. Scale bar 10 μm for all images. Right: Schematic of striatal regions where images were obtained. *CPu * caudate-putamen, *NAc*  nucleus accumbens

Animals were euthanized using deep anesthesia with isoflurane followed by decapitation (~ 4:00–6:00 PM) and their brains rapidly removed. Brains were post-fixed in 4% paraformaldehyde at 4° C for at least four days, cryoprotected in 30% sucrose solution in 0.1 M phosphate buffered-saline (PBS), and then sectioned coronally (40 μm) on a sliding freezing microtome. Brain sections were well preserved and there was no evidence of fixation issues such as retraction spaces surrounding neurons and other cells or weirdly cloudy and dark nissl staining of neurons (Garman et al. 1990). Brain sections were mounted onto gelatin coated slides, stained with cresyl violet (via Nissl staining) using a protocol adapted from a previous study (Cao and Patisaul 2011), coverslipped using Permount, and blinded to the experimenter for sex and estrous cycle phase (Fig. 1). Sections were imaged using Stereologer Software from SRC Biosciences on a Leica DM2500 P light microscope with a 63x objective coupled to a Imi-Tech IMC-147ft digital camera. Image files were saved in BMP file format. Given the distribution of soma sizes measured by this study and that 90–95% of striatal neurons are medium spiny neurons (Graveland & DiFiglia 1985; Tepper and Bolam 2004), the vast majority of the neurons measured in this study were medium spiny neurons (also called spiny projection neurons). Striatal regions were assessed between 2.28 and 0.84 bregma. Counting box localization followed a previous study (Meitzen et al. 2011). For the CPu, the counting boxes were situated in the dorsomedial and dorsolateral portions. For the NAc core, the analyzed region encompassed both the medial and lateral areas adjacent to the anterior commissure (Meitzen et al. 2011). For the NAc shell, the region analyzed was medial to the lateral ventricle.

### Morphometry

Morphometric methods followed those employed in a previous study (Meitzen et al. 2011), which were based upon protocols developed by Brenowitz and colleagues (Meitzen et al. 2007; Thompson and Brenowitz 2005; Tramontin et al. 1998). Methods employed were consistent with the principles of unbiased stereology. Neuronal density and soma size were measured in both hemispheres and along the rostral-caudal axis of the NAc core, NAc shell, and CPu using Fiji (Version 1.54f). Briefly, following a previous study (Meitzen et al. 2011), a large counting box (141.84 × 86.73 μm) was placed within the NAc core, NAc shell, and CPu in a systematic and random manner. A large counting box was used to minimize sampling variance by including both patch and matrix, following a previous study of striatal neuron density (Rosen and Williams 2001). Patch and matrix medium spiny neurons have similar morphology and intrinsic membrane properties (Kawaguchi et al. 1989, 1990). At least two of these large fields were counted in each hemisphere, and at least 200 neurons were counted per region per animal balanced across hemispheres. Neuronal nucleoli were used as the unit of count to obtain estimates of neuron density that were unbiased by soma size or shape. Nucleoli that were bisected by either the left boundary or the upper boundary of the counting frame were counted, whereas those that were bisected by the right boundary or the lower boundary were not counted as per unbiased counting techniques (Saper 1996). Along the z-axis, nucleoli were counted if they were bisected by the upper boundary but not the lower. To account for sectioning artifacts at the tissue edge (e.g., lost nucleolus caps), nucleoli in the top and bottom 7.5 μm of the section were not included in the count. The size of neuronal nucleoli does not differ between male and female rat striatal regions (Meitzen et al. 2011), so it is unlikely that splitting errors introduced asymmetric bias into the dataset (Guillery and Herrup 1997; Saper 1996). Density was calculated by dividing neuron count by the volume of the tissue sampled and averaging across sampling boxes. The reliability of our neuron density measurements was assessed by having a different experimenter blindly re-measure neuron density across select subjects. Neurons were distinguished from glia by the clear presence of a nucleolus, a well-defined nuclear envelope, nongranular cytoplasm, and/or an obvious axon hillock, as in previous studies (Markham et al. 2007; Meitzen et al. 2007; Rubinow and Juraska 2009; Tramontin et al. 1998). Measurements were made blind to treatment groups but not regions.

Methods for measuring neuron size also followed those previously employed (Meitzen et al. 2011). Neuron size was measured using the cross-sectional area of the soma using the tracing tool in Fiji (Version 1.54f). At least two large fields were assessed per hemisphere as described above, and a minimum of 100 neurons per region per animal were measured, balanced between hemispheres. This sample size was sufficient to encompass the entire range of variability in striatal neuron soma size (Meitzen et al. 2011). Neurons were distinguished from glia as described above, and all measurements were made blind to the treatment group but not region as described above.

*Statistics*.

Data was analyzed with unpaired two-tailed t tests using Welch’s correction, one-way ANOVA with Fisher’s LSD multiple comparison test, linear regressions or ANCOVA as appropriate (GraphPad Prism 6.07). Distributions were analyzed for normality with the Shapiro-Wilk normality test. P values < 0.05 were considered a priori as significant. Data are presented as mean ± SEM.

## Results

### Neuron soma size and density are different across striatal region irrespective of sex and estrous cycle phase

**Fig. 2:**
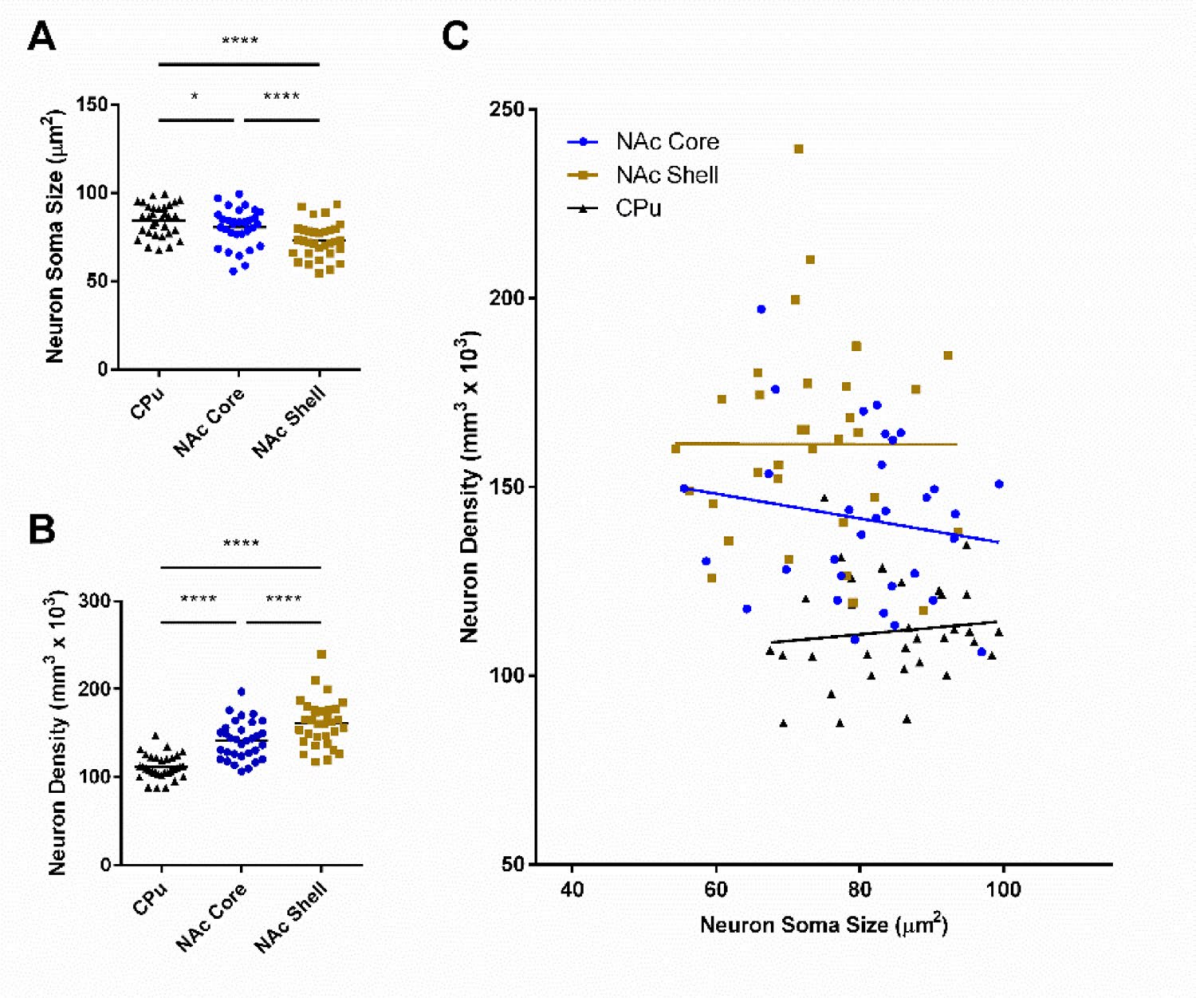
Differences exist in neuron soma size and density between striatal regions irrespective of sex or estrous cycle phase. **A** Neuron soma size differs by region, being largest in the CPu (84.6 ± 3.3 µm^2^), intermediate in the NAc core (80.5 ± 3.8 µm^2^), and smallest in the NAc shell (73.0 ± 3.6 µm^2^). **B** Neuron density differs by region, being most dense in the NAc shell (163.3 ± 9.5 neurons/mm^3^ × 10^3^), intermediate in the NAc core (141.5 ± 7.8 neurons/mm^3^ × 10^3^), and least dense in the CPu (111.8 ± 5.0 neurons/mm^3^ × 10^3^). **C** The linear regressions calculated for each region’s neuron density versus soma size differs by elevation (NAc shell *P* = 0.9936, R^2^ = 2.151e-006; NAc core *P* = 0.3834, R^2^ = 0.02542; CPu *P* = 0.5325, R^2^ = 0.01312). *CPu* caudate-putamen, *NAc* nucleus accumbens; * = *P* < 0.05; **** = *P* < 0.0001

The first hypothesis we tested was whether neuron density and soma size differed across striatal region independent of sex or estrous cycle phase. Neuron soma size differed by region (Fig. 2A; F_(2,31)_ = 25.78, *P* < 0.0001), and was largest in the CPu, intermediate in the NAc core, and smallest in the NAc shell (Post hoc tests: NAc core vs. NAc shell: t = 4.607, *P* < 0.0001; NAc core vs. CPu: t = 2.714, *P* = 0.0108; NAc shell vs. CPu: t = 6.539, *P* < 0.0001). Neuron density also differed by region (Fig. 2B; F_(2,31)_ = 79.42, *P* < 0.0001). The NAc shell featured the highest density, followed by the NAc core and then the CPu (Post hoc tests: NAc core vs. NAc shell: t = 4.745, *P* < 0.0001; NAc core vs. CPu: t = 8.855, *P* < 0.0001; NAc shell vs. CPu: t = 11.60, *P* < 0.0001). To further assess the robustness of these regional differences, we plotted neuron density versus soma size, performed linear regressions by region and then tested whether the lines differed using an ANCOVA (Fig. 2C). The slopes of each region’s individual linear regression did not differ from zero (NAc shell: *P* = 0.9936, Slope: -0.003947 ± 0.4913; NAc core: *P* = 0.3834, Slope: -0.3273 ± 0.3701; CPu: *P* = 0.5325, Slope: 0.1739 ± 0.2754). When the linear regressions are compared to each other, the elevations of the lines differed significantly between regions (F_(2,92)_ = 34.18, *P* < 0.0001). The slopes of the lines did not differ between regions (F_(2,92)_ = 0.419305, *P* = 0.6588). These data indicate that different striatal regions can be distinguished based upon neuron size and density.

### Nucleus accumbens shell: sex influences neuron soma size while estrous cycle does not

**Fig. 3:**
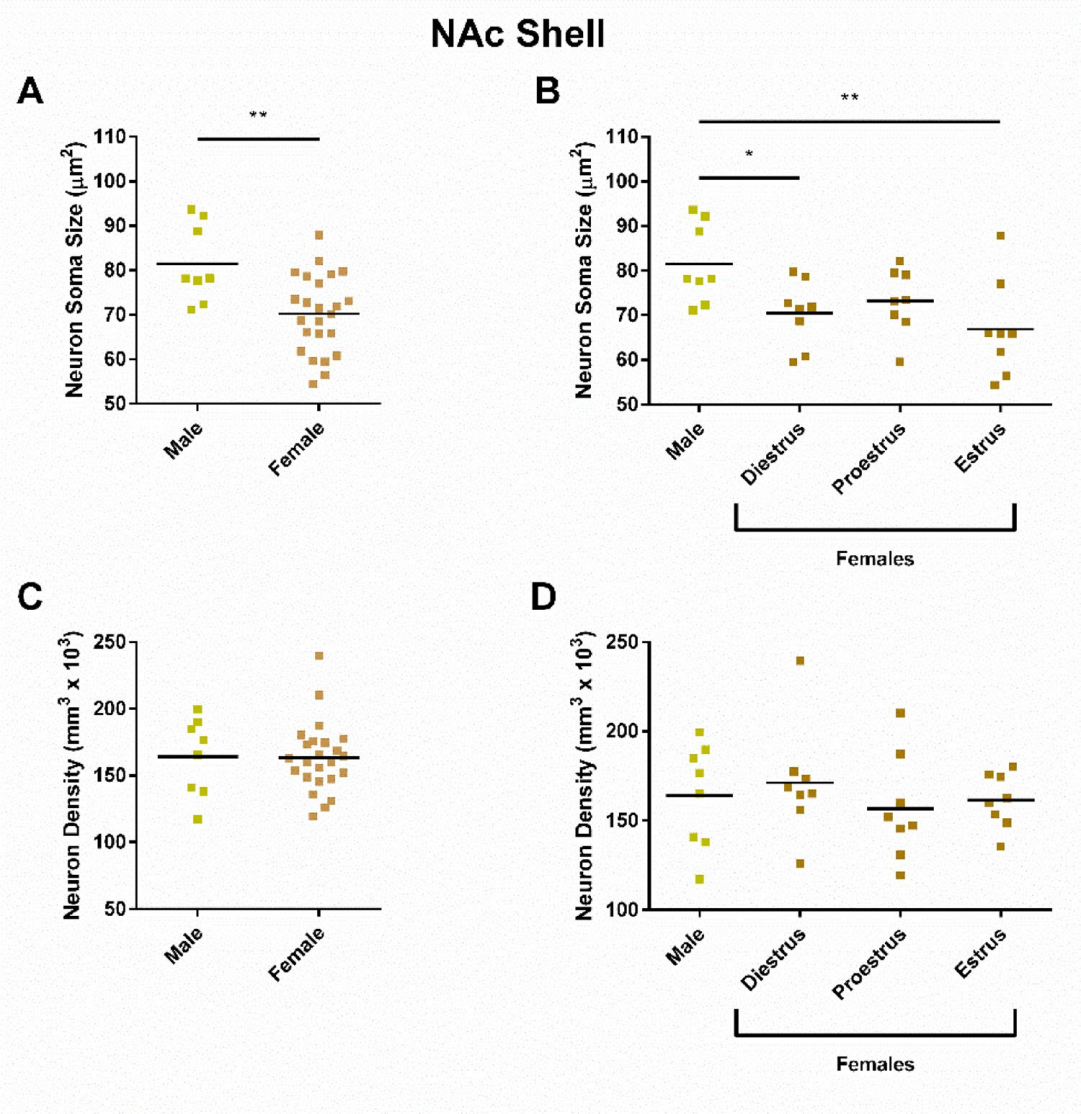
NAc shell soma size, but not neuron density differs by sex. **A** Soma sex analyzed by sex. Neuron soma size was smaller in females (70.2 ± 3.7 µm^2^) compared to males (81.5 ± 7.4 µm^2^). **B** Neuron soma size analyzed by estrous cycle phase. Neuron soma sizes were larger in males (81.5 ± 7.4 µm^2^) compared to diestrus phase (70.4 ± 6.2 µm^2^) and estrus phase (66.9 ± 9.2 µm^2^) but not proestrus phase (73.2 ± 6.1 µm^2^) females. **C** Neuron density did not differ by sex (Males: 164.0 ± 24.3, Females: 163.1 ± 11.0 neurons/mm^3^ × 10^3^). **D** Neuron density did not differ by estrous cycle phase (Diestrus: 171.3 ± 26.6, Proestrus: 156.6 ± 24.7, Estrus: 161.4 ± 12.7 neurons/mm^3^ × 10^3^). *NAc* nucleus accumbens; * = *P* < 0.05; ** = *P* < 0.01

In the NAc shell, neuron soma size was significantly different between males and females (Fig. 3A; t = 1.542, *P* = 0.0034), with males exhibiting larger soma sizes than females. Neuron soma size in males was significantly larger than that of females in the diestrus phase and estrus phase (Fig. 3B, F_(3,28)_ = 4.076, *P* = 0.0160; Post hoc tests: Diestrus vs. Males t = 2.542, *P* = 0.0168; Estrus vs. Males t = 3.349, *P* = 0.0023; Proestrus vs. Males t = 1.904, *P* = 0.0672; All other comparisons *P* > 0.15). No differences were found for neuron soma density by sex (Fig. 3C; t = 0.07873, *P* = 0.9378), or estrous cycle phase (Fig. 3D; F_(3,28)_ = 0.4069, *P* = 0.7492).

### Nucleus accumbens core: no differences across sex or estrous cycle phase

**Fig. 4:**
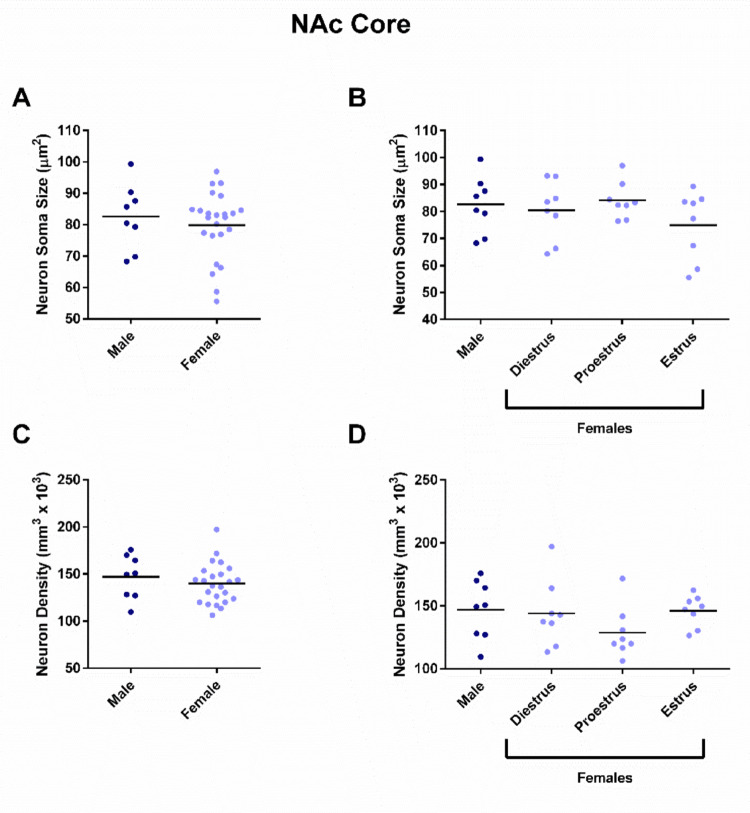
NAc core neuron soma size or density did not differ by sex or estrous cycle phase. **A** Neuron soma size did not differ by sex (Male: 82.6 ± 8.7, Female 79.8 ± 4.5 µm^2^). **B** Neuron soma size did not differ by estrous cycle phase (Diestrus: 80.5 ± 9.0, Proestrus: 84.1 ± 5.7, Estrus: 75.0 ± 10.7 µm^2^). **C** Neuron density did not differ by sex (Males: 146.9 ± 19.6, Females: 139.7 ± 8.9 neurons/mm^3^ × 10^3^). **D** Neuron density did not differ by estrous cycle phase (Diestrus: 144.1 ± 22.2, Proestrus: 128.9 ± 16.9, Estrus: 146.1 ± 10.3 neurons/mm^3^ × 10^3^). *NAc* nucleus accumbens

Regarding the NAc core, neuron soma size did not differ by sex (Fig. 4A; t = 0.6354, *P* = 0.5300) or estrous cycle phase (Fig. 4B; F_(3,28)_ = 1.193, *P* = 0.3304). No difference in neuron soma density was detected by sex (Fig. 4C; t = 0.8111, *P* = 0.4237) or estrous cycle phase (Fig. 4D; F_(3,28)_ = 1.274, *P* = 0.3024).

### Caudate-putamen: no differences across sex or estrous cycle phase

**Fig. 5:**
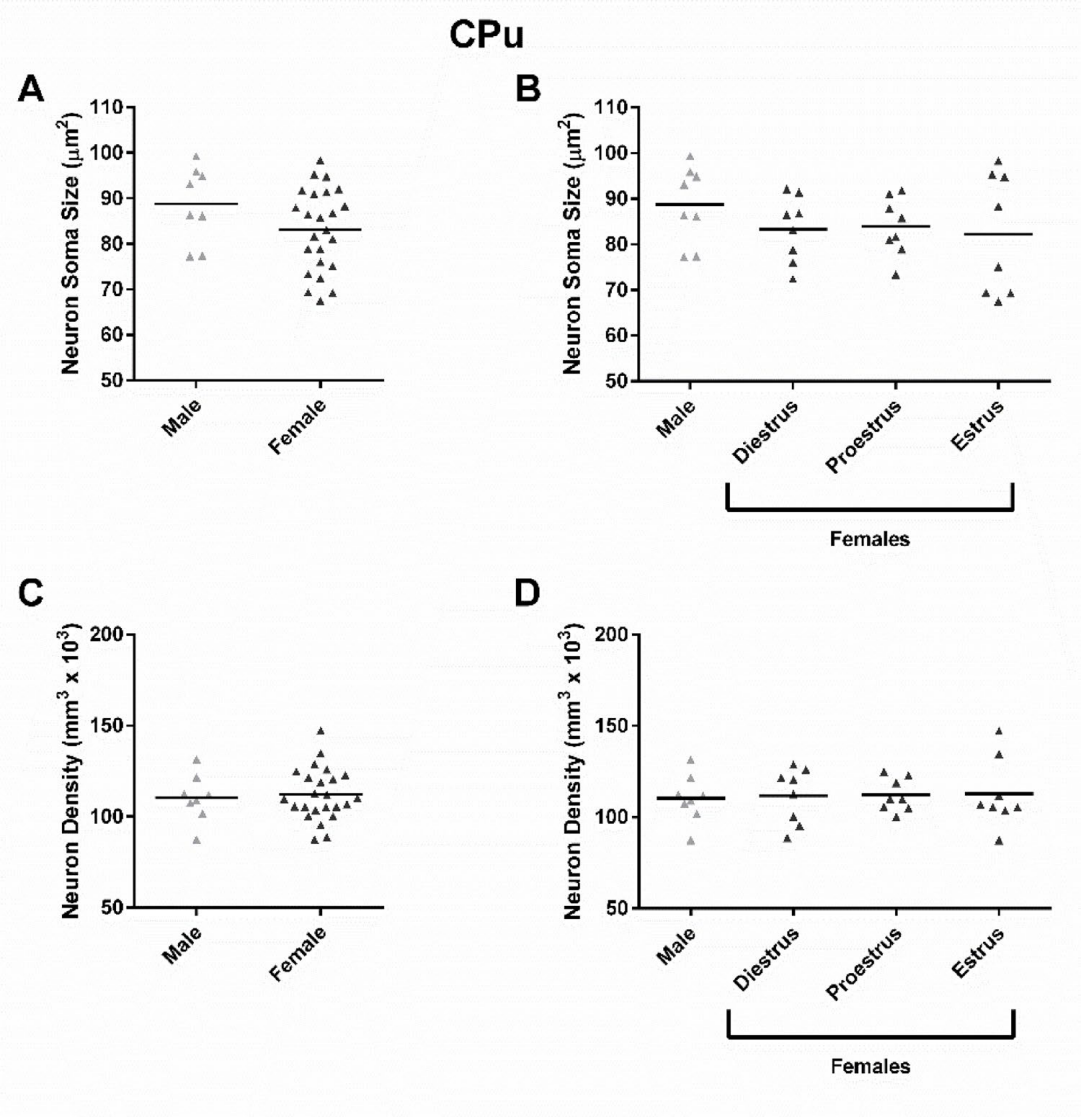
CPu neuron soma size or density did not differ by sex or estrous cycle phase. **A** Neuron soma size did not differ by sex (Male: 88.8 ± 7.0, Female 83.2 ± 3.8 µm^2^). **B** Neuron soma size did not differ by estrous cycle phase (Diestrus: 83.4 ± 5.9, Proestrus: 83.9 ± 5.3, Estrus: 82.2 ± 11.1 µm^2^). **C** Neuron density did not differ by sex (Males: 110.3 ± 10.8, Females: 112.2 ± 6.0 neurons/mm^3^ × 10^3^). **D** Neuron density did not differ by estrous cycle phase (Diestrus: 111.7 ± 12.6, Proestrus: 112.1 ± 7.5, Estrus: 112.8 ± 15.9 neurons/mm^3^ × 10^3^). *CPu* caudate-putamen

In the CPu, neuron soma size did not differ by sex (Fig. 5A; t = 1.542, *P* = 0.1337) or estrous cycle phase (Fig. 5B; F_(3,28)_ = 0.7896 *P* = 0.5099). Neuron soma density likewise did not differ by sex (Fig. 5C; t = 0.3251, *P* = 0.7473) or estrous cycle phase (Fig. 5D; F_(3,28)_ = 0.04105, *P* = 0.9887).

## Discussion

These experiments constitute the first quantification of striatal neuron size and density disaggregated by region, sex and estrous cycle phase in any animal. This work indicates regional differences between each of the striatal regions within gonad-intact rats as well as detecting a sex difference in neuron soma size in the NAc shell. No sex differences were detected in the NAc core and CPu. Additionally, there were also no significant differences across estrous cycle phase in any of the regions measured. These results expand upon previous work conducted in gonadectomized animals where sex differences in neuron size or density were also not observed (Meitzen et al. 2011), previous studies of striatal neuron morphometry where animal sex was not assessed as an experimental variable (Meredith et al. 1992), as well as striatal neuron dendrite anatomy studies where sex was included as a variable (Forlano and Woolley 2010; Wissman et al. 2011, 2012; Beeson and Meitzen 2023). Here we will first discuss these findings in the context of striatal region, then biological sex, and finish with the estrous cycle.

### Regional differences in striatal neuron soma size and density

Neuron soma size and density varied between the CPu, NAc core, and NAc shell regions irrespective of estrous cycle phase and sex. Indeed, neuron soma size was largest in the CPu compared to the NAc core and then NAc shell. Conversely, neuron density was greatest in the NAc shell, followed by the NAc core and then the CPu. These results largely align with previous studies (Rosen and Williams 2001; Meitzen et al. 2011), providing an important extension as well as validation. Somewhat counterintuitively, within each of the striatal regions, the relation between soma size and soma density was the same and did not correlate, even though the elevations of the linear regression calculated for each region differed. Thus, attributes other than or in addition to soma size likely mediate the regional differences in striatal neuron density. These regional changes in density could possibly be explained by cell number, dendritic arborization (Meredith et al. 1992), region-specific striosome and matrix compositions (Brimblecombe and Cragg 2017), differences in glia or vasculature, synaptogenesis, or myelination, including the size of the myelinated fibers emanating from the internal capsule that pass through these regions. Regarding cell number, a limitation of the current study is that striatal region volume was not assessed, which makes it impossible to estimate cell number via unbiased stereological cell counting techniques. Instead this study assessed neuron density, which can be accurately assessed without total region volume. Cell number as well as striatal region volume should be addressed in future studies. To our knowledge, rodent striatal region volume has never been assessed in the context of the estrous cycle, with previous experiments focusing on gonadectomized animals or not disaggregating data by sex (reviewed in Wong et al. 2016). In humans, preclinical neuroimaging studies have noted menstrual-cycle impacts on functional connectivity and other related metrics in striatal regions (Hidalgo-Lopez et al. 2020), suggesting that the rodent estrous cycle is likewise an important variable to assess.

Focusing on dendritic arborization, several studies have found that NAc shell neurons exhibit less extensive dendritic arbors compared to neurons in the NAc core (Forlano and Woolley 2010; Meredith et al. 1992; O’Donnell and Grace 1993). Striatal dendritic arborization is understudied in the context of sex and should be investigated further, especially since testosterone modulates this attribute (Wallin-Miller et al. 2016). Fewer studies have compared soma attributes between these subregions. For example, Meredith and colleagues did not detect a difference in soma size between NAc core and NAc shell (Meredith et al. 1992), unlike the current study and our laboratory’s previous work (Meitzen et al. 2011). We suspect this inconsistency is explained by Meredith’s smaller sample size and potentially the dehydrating effects of the golgi stain method upon somas. Interestingly, a recent paper from Maria-Rios and colleagues (Maria-Rios et al. 2023) found that male rat NAc shell neurons exhibited a lower electrophysiological capacitance concomitant with increased input resistance compared to NAc core. This result is consistent with our finding that the NAc shell neurons have smaller soma areas compared to those in the NAc core, as capacitance typically directly relates to neuron membrane surface area.

#### A sex difference in neuron soma size but not density in the NAc shell; no other region shows sex differences

Neuron soma size and density have long been a first assessed metric for potential sex differences across many different species and brain regions (Ball and Macdougall-Shackleton 2001; Cooke 2006; Nottebohm and Arnold 1976), and it is surprising that striatal soma size has never been assessed in the context of gonad-intact males and females. A priori, we did not expect to find sex differences in soma size in the NAc shell. This erroneous a priori expectation was based upon the significant influence of the estrous cycle and its component hormones on striatal neuron properties (Beeson and Meitzen 2023; Proaño et al. 2024). In contrast to our a priori expectation, our study detected a sex difference in neuron soma size within the NAc shell but not NAc core or CPu. Our findings specifically indicate a larger soma size for males within the NAc shell when compared to females across multiple estrous cycle phases. This is a highly interesting finding, begging the question of why the shell region demonstrates this sex difference.

While we cannot answer this question, we can place it within the broader framework of sex differences and similarities in the NAc shell and other striatal regions. Regarding electrophysiological properties, only prepubertal NAc shell medium spiny neurons have been assessed and they do not differ by sex during that developmental period (Willett et al. 2016). Adult NAc shell neuron electrophysiological properties have never, to our knowledge, been comprehensively assessed for sex differences or estrous cycle impacts and this gap in knowledge should be addressed (Krentzel et al. 2022). Other, more recent, findings also hold relevance. Sex differences have been described in D1 dopamine receptor-expressing medium spiny neurons in the NAc shell (Aziz and Mangieri 2024), however capacitance and related features were not assessed so it is challenging to relate the findings directly back to soma membrane area. There has also been a sex difference in how local field potential oscillatory activity relates to hedonic processing (Douton and Carelli 2023). Another recent paper discovered sex differences in cocaine-induced neuronal excitability in D2 versus D1 expressing medium spiny neurons (Chapp et al. 2024). There have also been sex differences described in select glutamatergic inputs into the NAc shell (Johnson et al. 2023). Overall, this preponderance of evidence indicates that the NAc shell is sexually differentiated, and that sex as an experimental variable in gonad-intact animals will be an important consideration for future experiments in this brain region. This focus upon gonad-intact animals is intentional. In the previous study of gonadectomized rats, a sex difference in NAc shell neuron soma size was not detected (Meitzen et al. 2011). While there are several possibilities as to why a sex difference was detected in the current versus this previous study (including experimental sample number and rat strain), the biggest difference between the two studies is the use of gonad-intact compared to gonadectomized rats. It is now established that androgens and estrogens sex-specifically influence NAc neuron dendritic spines in both males and females, that this region expresses nuclear-localized sex steroid hormone receptors during development and membrane-localized sex steroid hormone receptors in adulthood, and that neurosteroids including estrogens and androgens generally modulate the larger mesocorticolimbic system (Peterson et al. 2015; Bradshaw et al. 2024; Staffend et al. 2011; Gross et al. 2018; Huijgens et al. 2021; Krentzel et al. 2021; Wallin-Miller et al. 2016; Seib et al. 2023). During early development Estrogen Receptor α shows sex-specific expression in gonad-intact rat NAc, however by adulthood there is little evidence of sex differences in estrogen and androgen receptors. Importantly, hormone receptor expression has not been rigorously assessed in either pubertal or adult rodent striatal regions across estrous and testosterone cycles (Almey et al. 2012, 2016, 2022; Krentzel et al. 2021; Quigley and Becker 2021). It is also unknown how gonadectomy may have altered hormone receptor expression levels. What is certain is that gonadectomy eliminated a chronic source of hormonal support, including testosterone and its androgenic and estrogenic metabolites in males, that perhaps likewise eliminated the sex difference in soma size. Evaluating the role of gonad-sourced testosterone and its neurosteroid metabolites in the NAc shell will be an important avenue for future research.

#### No differences detected across estrous cycle phases in neuron soma size or density

This study further explored whether estrous cycle phase modulates striatal neuron morphology, extending our laboratory’s previous study of striatal neuron spine shape and density (Beeson and Meitzen 2023). We did not detect differences for soma size and density across estrous cycle phase within female animals, unlike the estrous cycle impacts upon dendritic spine shape (Beeson and Meitzen 2023). This finding from our study confirms that the sex difference in soma size in the NA shell is present across multiple estrous cycle phases, consistent with the conclusion that this difference in soma size is related to sex and not driven by the estrous cycle. We are unaware of documentation of changes or lack of changes in neuron soma size over the course of the estrous cycle in any other brain region, as the majority of studies focus on dendritic attributes such as spine density and shape dating since Woolley and colleagues’ initial study in the hippocampus (Woolley et al. 1990). There is evidence of soma size differing by sex (cited above), and between reproductive state in adults of select species that breed seasonally (Amorin and Calisi 2015). However, these seasonal changes are relatively slow, and even when intentionally facilitated via hormone and photoperiod exposure in the laboratory, the changes in soma size occur over the time course of 3 to 7 days (Meitzen et al. 2007; Thompson and Brenowitz 2010). In contrast, the rat estrous cycle typically presents a 4-to-5-day time course, with phases such as proestrus only lasting for a matter of hours. Thus, while it was not outside the realm of possibility that a short estrous cycle phase could change soma size, overall, we found no evidence that the estrous cycle regulates soma size and density. This may be because dendritic spine shape and density is simply much more plastic than soma size and number. This lack of detectable changes in neuron soma size and density over the estrous cycle argues that modulation of other factors play an important role in mediating impacts on striatal anatomy, function, and ultimately relevant behaviors and disorders.

## Data Availability

The datasets generated during and/or analyzed during the current study are available in the Dryad repository (10.5061/dryad.zkh1893nk).
